# PROTAC induced-BET protein degradation exhibits potent anti-osteosarcoma activity by triggering apoptosis

**DOI:** 10.1038/s41419-019-2022-2

**Published:** 2019-10-25

**Authors:** Chengcheng Shi, Huapeng Zhang, Penglei Wang, Kai Wang, Denghui Xu, Haitao Wang, Li Yin, Shuijun Zhang, Yi Zhang

**Affiliations:** 1 Department of Pharmacy, The First Affiliated Hospital of Zhengzhou University, Zhengzhou, Henan China; 2 Department of Hepatobiliary and Pancreatic Surgery, The First Affiliated Hospital of Zhengzhou University, Zhengzhou, Henan China; 3 Open and Key Laboratory of Hepatobiliary & Pancreatic Surgery and Digestive Organ Transplantation at Henan Universities, Zhengzhou, Henan China; 4 Henan Key Laboratory of Digestive Organ Transplantation, Zhengzhou, Henan China; 5 Zhengzhou Key Laboratory of Hepatobiliary & Pancreatic Diseases and Organ Transplantation, Zhengzhou, Henan China; 6 China-US (Henan) Hormel Cancer Institute, Zhengzhou, Henan China; 7 Department of Orthopaedic Surgery, The First Affiliated Hospital of Zhengzhou University, Zhengzhou, Henan China

**Keywords:** Bone cancer, Target identification, Bone cancer

## Abstract

Targeting oncogenic proteins for degradation using proteolysis-targeting chimera (PROTAC) recently has drawn increasing attention in the field of cancer research. Bromodomain and extra-terminal (BET) family proteins are newly identified cancer-related epigenetic regulators, which have a role in the pathogenesis and progression of osteosarcoma. In this study, we investigated the in vitro and in vivo anti-osteosarcoma activity by targeting BET with a PROTAC molecule BETd-260. The results showed that BETd-260 completely depletes BET proteins and potently suppresses cell viability in MNNG/HOS, Saos-2, MG-63, and SJSA-1 osteosarcoma cell lines. Compared with BET inhibitors HJB-97 and JQ1, the activity of BETd-260 increased over 1000 times. Moreover, BETd-260 substantially inhibited the expression of anti-apoptotic Mcl-1, Bcl-xl while increased the expression of pro-apoptotic Noxa, which resulted in massive apoptosis in osteosarcoma cells within hours. In addition, pro-oncogenic protein c-Myc also was substantially inhibited by BETd-260 in the OS cells. Of note, BETd-260 induced degradation of BET proteins, triggered apoptosis in xenograft osteosarcoma tumor tissue, and profoundly inhibited the growth of cell-derived and patient-derived osteosarcoma xenografts in mice. Our findings indicate that BET PROTACs represent a promising therapeutic agent for human osteosarcoma.

## Introduction

Osteosarcoma (OS) is the most common type of primary malignant bone tumor, predominantly affecting the health and life of children and young adults^1^. Current treatment measures for OS include surgical resection and adjuvant chemotherapy. These conventional treatments offer a significant survival advantage in patients with localized OS^2^. Unfortunately, patients with metastasized or recurrent OS often do not respond adequately to these standard treatments. Therefore, it is imperative to explore novel agents and new therapeutic approaches for OS treatment^2,3^. Recently, epigenetic dysregulation and its role in pathogenesis and cancer progression have drawn increasing attention^4–7^. Bromodomain and extra-terminal (BET) family proteins are newly identified cancer-related epigenetic regulators, which are critical for sustaining the expression of numerous oncogenes^6–8^. BET family member bromodomain-containing protein 4 (BRD4) has been found to be highly expressed in OS tumor tissues and OS cell lines, and may play an important role in the development of aggressive OS^9–11^. Suppression of the biological function of BET proteins with small molecule BET inhibitor JQ1 as a single agent or in combination with other anticancer agents leads to anti-proliferative activity and modestly inhibits OS xenograft tumor growth^9–11^. This suggests that targeting BET may have beneficial effects for OS patients.

**Fig. 1:**
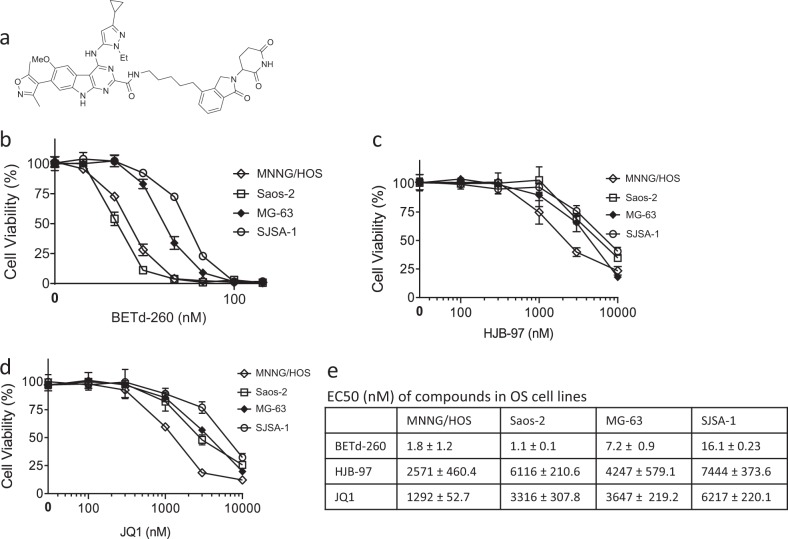
BETd-260 displays potent activity in suppressing cell viability in OS cells. **a** The structure of BETd-260 were shown. OS cell lines Saos-2, MNNG/HOS, MG-63, and SJSA-1 were treated with serially diluted BETd-260 (**b**), HJB-97 (**c**), or JQ1 (**d**) as indicated for 72 h. Cell viability was examined by CCK-8 assay. **e** The EC50 values were calculated with Prism 6 software and listed. Data are mean ± SEM (*n* = 4)

BET protein proteolysis-targeting chimera (BET PROTACs) are bifunctional molecules with one side bound to BET proteins and the other side recognized by the Cullin-dependent E3 ligase. By hijacking the ubiquitin-proteasome system (UPS), BET PROTACs selectively and completely induce degradation of BET proteins in cancer cells, and thus represent a more efficient strategy for targeting BET proteins^12–15^. In the present study, we evaluated the anti-OS potential of BET PROTACs and investigated the possible underlying molecular mechanisms. For this purpose, we used a small molecule, BET PROTAC BETd-260 with purity of 99.35% (Fig. 1a). BETd-260 was synthesized based on BET inhibitor HJB-97, and had good pharmacological activities and pharmacokinetic properties^14,15^. Our results showed that by degrading multiple BET members, and by modulating the expression of Bcl-2 family members, BETd-260 triggered massive apoptosis in OS cells and in OS xenograft tumor tissue, and eventually led to profound and sustained inhibition of tumor growth in both OS cell-line-derived xenografts and patient-derived xenografts (PDX) models in mice.

## Results

### BETd-260 displays potent abilities to suppress cell viability in OS cells

We first evaluated the biological activity of BETd-260 by CCK-8 assay in a panel of four OS cell lines: Saos-2, MNNG‐HOS, MG‐63, and SJSA-1 cell lines. The results showed that BETd-260 potently and dose-dependently inhibited cell viability in all four OS cell lines with low nanomolar EC_50_ values. MNNG/HOS and Saos-2 were exceptionally sensitive to the treatment. BETd-260 had EC_50_ values of 1.8 and 1.1 nmol/L, respectively, in these two cell lines. Consistent with the previous findings of Baker et al.^11^, BET inhibitors JQ1 and HJB-97 had only modest activity in the inhibition of cell viability, with 1292–7444 nmol/L EC_50_ values in the four cell lines (Fig. 1b–e). Notably, both BET inhibitors at 3000 nmol/L did not completely inhibit cell viability in the OS cells (Fig. 1c–e). In striking contrast, BETd-260, even at a dose as low as 10–100 nmol/L, completely eliminated the cell viability in the OS cell lines (Fig. 1b).

### BETd-260 is a potent BET degrader in OS cells

**Fig. 2:**
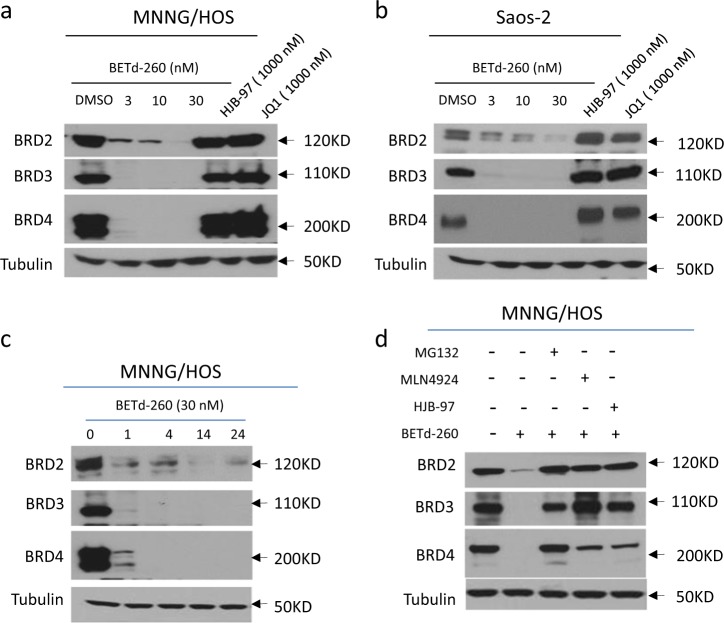
BETd-260 potently induces degradation of BET proteins in OS cells. **a** MNNG/HOS and **b** Saos-2 cell lines were treated with BETd-260, HJB-97, or JQ1 as indicated for 24 h. The protein levels of BRD2, 3, and 4 were examined by western blotting analysis. Tubulin was used as a loading control. **c** The MNNG/HOS cell line was treated with BETd-260 at 30 nM for different times. The protein level of BRD2, 3, and 4 were examined by western blotting analysis. Tubulin was used as a loading control. **d** MNNG/HOS cells were pretreated with MG-132 (2000 nmol/L), MNL4924 (500 nmol/L), or HJB-97 (3000 nmol/L) for 1 h, followed by treatment with BETd-260 (10 nM) for 2 h. The protein levels of BRD2, 3, and 4 were examined by western blotting analysis. Tubulin was used as a loading control. Data are representative of three independent experiments

We then examined the activity of BETd-260 in degrading BET proteins in the OS cell lines. Western blotting results showed that BETd-260 potently degraded BRD2/3/4 in all four OS cell lines (Fig. 2a, b and SI Fig. 1a,b). Of note, BETd-260 at 3 nmol/L for 24 h completely depleted BRD3 and 4, and largely suppressed the level of BRD2 protein in MNNG/HOS and Saos-2 cell lines (Fig. 2a, b). Time course analysis showed that BETd-260 at 30 nmol/L achieved maximum degradation effect within 1 h in the MNNG/HOS cells, and this strong effect lasted up to 24 h (Fig. 2c). Consistent with data reported previously^11^, BET inhibitors JQ1 and HJB-97 had no effect on the levels of three BET proteins in OS cells.

In western blotting assays, we found that proteasome inhibitor MG-132 induced accumulation of BET proteins at both ubiquitinated and basal levels in MNNG/HOS cell line (SI Fig. 1c). These data indicated that BET proteins in OS cells were subject to constitutive UPS-dependent degradation. We further investigated whether BET protein degradation triggered by BETd-260 also was through UPS-dependent pathway by MG-132 and MLN4924, a pan-inhibitor of Cullin-based E3 ligase. The results of western blotting assays showed that pre-treatment with either MG-132 or MLN4924 largely abrogated the ability of BETd-260 to degrade BET proteins in MNNG/HOS cells (Fig. 2d). Moreover, pre-treatment with excess amount of HJB-97 (10,000 nmol/L) also effectively inhibited BETd-260 to degrade BET proteins. These results are in agreement with the notion that BETd-260 was designed to induce selective degradation of BET proteins through Cullin4A mediated-UPS and HJB-97-mediated specific binding of BET proteins^14^.

### BETd-260 triggers massive apoptosis in OS cells

**Fig. 3:**
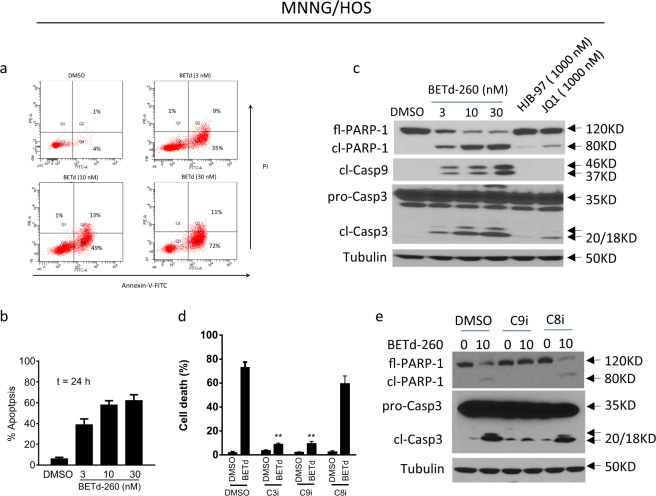
BETd-260 triggers massive apoptosis in the MNNG/HOS cell line. **a**, **b** The MNNG/HOS cell line was treated with BETd-260 as indicated for 24 h. **a** Apoptosis was examined by propidium iodide (PI)/Annexin-V staining in combination with flow cytometry assay. **b** Average percentages of apoptosis from three independent experiments were plotted in the graphs. **c** The MNNG/HOS cell line was treated with BETd-260, HJB-97, or JQ1 as indicated for 24 h. The cells were lysed and the protein levels of full-length PARP-1 (fl-PARP-1), cleaved PARP-1 (cl-PARP-1), cleaved caspase-9 (cl-Casp9), pro-Caspase-3 (pro-Casp3), and cleaved caspase-3 (cl-Casp3) were examined by western blotting analysis. Tubulin was used as a loading control. **d** MNNG/HOS cells were pretreated with caspase-3 inhibitor Z-DEVD-FMK (C3i, 10,000 nmol/L), caspase-9 inhibitor Z-LEHD-FMK (C9i, 10,000 nmol/L), or caspase-8 inhibitor Z-IETD-FMK (C8i, 10,000 nmol/L) for 1 h, followed by treatment with BETd-260 (10 nM) for 48 h. Cell death induction was examined by Trypan blue exclusion assay. **e** MNNG/HOS cells were pretreated with C9i (10,000 nmol/L) or C8i (10,000 nmol/L) for 1 h, followed by treatment with BETd-260 (10 nM) for 24 h. The levels full-length PARP-1 (fl-PARP-1), cleaved PARP-1 (cl-PARP-1), pro-Caspase-3 (pro-Casp-3), and cleaved Caspase-3 (cl-Casp-3) were examined by western blotting. Tubulin was used as a loading control. Data are representative of three independent experiments

We next investigated the apoptotic activity of BETd-260 in OS cells. After treatment with BETd-260 overnight, a large fraction of OS cells became shrunken, round and floating, consistent with the cell morphology alterations of apoptosis. We then used Annexin-V-FITC/PI-labeling flow cytometry to confirm this apoptotic effect. As shown in Fig. 3a, b, treatment with BETd-260 at 3, 10, and 30 nM for 24 h induced apoptosis in 43%, 62%, and 84% of the MNNG/HOS cell line, respectively (Fig. 3a, b). The same treatments induced apoptosis in 25%, 57%, and 75% of the Saos-2 cell line, respectively (SI Fig. 2a, b). Western blotting revealed that BETd-260 induced activation of caspase-3, -9 and cleavage of poly (ADP-ribose) polymerase (PARP-1) in both OS cell lines (Fig. 3c and SI Fig. 2c), suggesting the involvement of caspase activity in the apoptosis. We used specific pharmaceutical caspase inhibitors to investigate the role of caspase activation in BETd-260-mediated apoptotic activity. The results showed that caspase-3 (Z-DEVD-FMK) and -9 (Z-LEHD-FMK) inhibitors almost completely abrogated the ability of BETd-260 to induce cell death, while caspase-8 (Z-IETD-FMK) inhibitor had a modest effect on the cell death induction in MNNG/HOS and Saos-2 cell lines (Fig. 3d and SI Fig. 2d). Moreover, caspase-9 (Z-LEHD-FMK) inhibitor largely attenuated BETd-260-triggered PARP-1 cleavage and caspase-3 activation, whereas caspase-8 (Z-IETD-FMK) inhibitor had very weak effect on the apoptosis signaling in the two OS cell lines (Fig. 3e and SI Fig. 2e). These results demonstrated that apoptosis triggered by BETd-260 in OS cells relied on the caspase-9 and -3 cascade-mediated intrinsic pathway.

In contrast, BET inhibitors JQ1 and HJB-97 at 1000 nM triggered no or modest activation of apoptosis signaling in OS cells (Fig. 3c and SI Fig. 2c). These findings suggest that BET inhibitors have weak apoptotic activity in OS cells, consistent with previous findings in other solid cancers^12,15^.

### BETd-260 modulates the expression of three Bcl-2 family members and inhibits the expression of c-Myc in OS cells

**Fig. 4:**
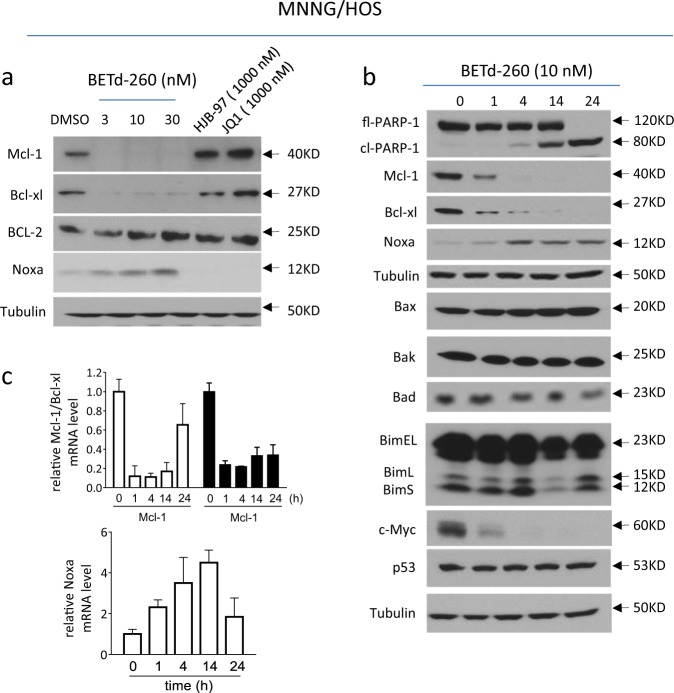
BETd-260 modulates the expression of Bcl-2 family members in OS cells. **a** MNNG/HOS cell line was treated with BETd-260, HJB-97, or JQ1 as indicated for 24 h. The levels of Mcl-1, Bcl-xl, Bcl-2, and Noxa were examined by western blotting analysis. Tubulin was used as a loading control. **b** The MNNG/HOS cell line was treated with BETd-260 (10 nmol/L) for 1, 4, 14, and 24 h. The levels of fl-PARP-1, cl-PARP-1, Mcl-1, Bcl-xl, Noxa, Bax, Bak, Bad, three isoforms of Bim (BimEL, BimL, and BimS), c-Myc and p53 proteins in the treated cells were examined by western blotting analysis. Tubulin was used as a loading control. **c** The mRNA levels of Mcl-1, Bcl-xl, and Noxa in the treated cells were examined by RT-PCR assay. Data are representative of three independent experiments

Because the Bcl-2 family of proteins plays a key role in the initiation and execution of apoptosis, we next investigated whether the robust apoptotic activity of BETd-260 in OS cells was associated with the modification of these key apoptotic proteins. We first treated the OS cells with BETd-260 and examined the protein levels of several key Bcl-2 family members by western blotting (Fig. 4a, b and SI Fig. 3a, b). In agreement with previous findings in triple negative breast cancer with BET PROTAC BETd-246^15^, BETd-260 rapidly caused complete depletion of Mcl-1 in OS cells. BETd-260 also eliminated another anti-apoptotic protein, Bcl-xl. In contrast, the expression levels of Bcl-2 itself, and the levels of pro-apoptotic Bcl-2 family members Bad and Bim remained mostly unchanged after BETd-260 treatment (Fig. 4b and SI Fig. 3b). In addition, the results also showed that BET inhibitors JQ1 and HJB-97 had no or little effect on the levels of these important apoptotic proteins in the OS cells (Fig. 4a and SI Fig. 3a).

The results of western blotting further showed that BETd-260 increased the mRNA and protein levels of pro-apoptotic Noxa in MNNG/HOS and Saos-2 cell lines in a dose-dependent manner (Fig. 4a–c and SI Fig. 3a, b). Silencing Noxa significantly inhibited cell death in the MNNG/HOS cell line (SI Fig. 4), suggesting a role of Noxa in apoptosis induction.

The results of western blotting also indicated that BETd-260 had no or little impact on the levels of Bax and Bak (Fig. 4b and SI Fig. 3b). Nevertheless, since it was reported that Bax/Bak executes their pro-apoptotic function through conformational change (oligomerization) at the level of the mitochondria^16^, we thus investigated the role of Bax/Bak in BETd-260-mediated cell death by simultaneously knocking down of Bax and Bak. The results showed that BETd-260-mediated cell death and PARP-1 cleavage were significantly attenuated when the expression of Bax/Bak was inhibited by siRNAs in OS cells (SI Fig. 5). These results suggest that Bax/Bak play an essential role in BETd-260-mediated anti-OS activity, although the basal level of Bax/Bak was not obviously altered (SI Fig. 5).

Previous studies have shown that BET degraders inhibited c-Myc in breast cancer and prostate cancer cells, and that p53 was involved in the anticancer activity mediated by BET inhibitor JQ1 in acute myeloid leukemia and medulloblastoma cells^17,18^. We next investigated the effect of BETd-260 on c-Myc and p53 in OS cells. The results showed that BETd-260 treatment distinctly reduced the level of c-Myc protein in three OS cell lines. Moreover, the reduction occurred as early as 1 h, and last to 24 h (Fig. 4b and SI Fig. 3b). These results suggest that c-Myc is an important target of the BET degrader in OS cells. However, the level of p53 protein remained unchanged after BETd-260 treatment for 24 h either in MNNG/HOS, a cell line with p53 mutation or SJSA-1, a cell line harboring a wild-type p53 gene (Fig. 4b and SI Fig. 3b). These results suggest that p53 may not be involved in the BETd-260-mediated anticancer activity in these OS cell lines.

### BETd-260 induces BET degradation, triggers massive apoptosis in vivo, and inhibits tumor growth in MNNG/HOS xenografts in mice

**Fig. 5:**
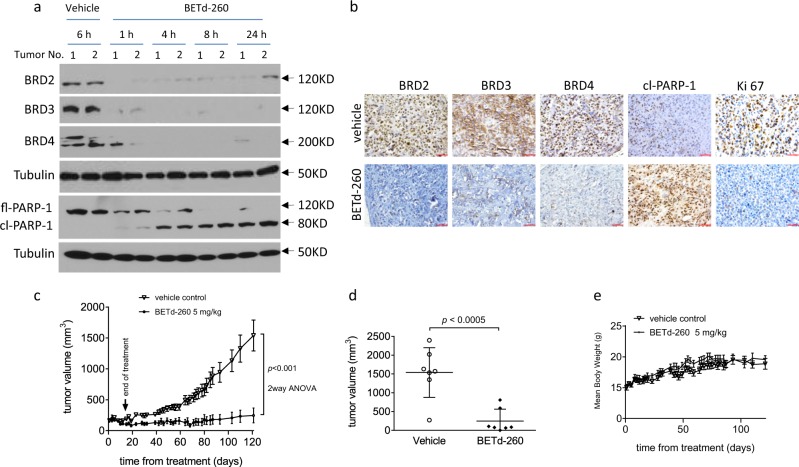
BETd-260 induces BET degradation, triggers apoptosis in vivo, and inhibits tumor growth in MNNG/HOS xenografts in mice. **a**, **b** BALB/c mice bearing MNNG/HOS xenograft tumors were treated with a single intravenous dose of 5 mg/kg BETd-260. Two to three mice were sacrificed and tumor tissue was harvested at each different time points. **a** The levels of BRD2, BRD3, and BRD4 proteins in tumor tissue lysates were examined by western blotting analysis. Tubulin was used as a loading control. **b** The expression of BRD2, BRD3, BRD4, cleaved PARP-1, and Ki67 was examined by immunohistochemistry staining. **c**–**e** BALB/c mice bearing xenograft tumors were treated with BETd-260 and vehicle control intravenously three times per week for 3 weeks. Tumor sizes and body weights were measured every 2–3 days. Data are mean ± SEM (*n* = 7–8). **c** Tumor values were plotted with Prism 6 software. **d** Scatter plots represent the tumor volumes at the end of each study. **e** Body weights were plotted with Prism 6 software. *p* values between each treated and the vehicle control group were determined using two-way ANOVA. *p < 0.05; **p < 0.01; ***p < 0.001; ****p < 0.0001)

We investigated whether BETd-260 degraded BET proteins and induced apoptosis in OS tumor tissues by performing a pharmacodynamic (PD) study in MNNG/HOS OS xenograft tumor tissue. Mice bearing MNNG/HOS xenograft tumors were treated with a single intravenous dose of BETd-260 at 5 mg/kg and two to three mice were sacrificed at 1, 4, 8, and 24 h time points, respectively. After the treatment, xenograft tumors were harvested. Western blotting showed that BETd-260 treatment completely depleted BRD2, BRD3, and BRD4 proteins in the tumor tissue (Fig. 5a). The depletion started from 1 h after treatment and lasted more than 24 h. These findings were validated by IHC staining analysis (Fig. 5b). Moreover, BETd-260 treatment also triggered PARP-1 cleavage, which was validated by IHC assays showing that a large amount of cells were positive for cleaved PARP-1 in BETd-260-treated tumors, but not in the control tumors, suggesting that BETd-260 treatment resulted in massive apoptosis in OS tumor tissue (Fig. 5a, b). IHC staining further showed that after treatment for 24 h, a large fraction of tumor cells became Ki67-negative (Fig. 5b and SI Fig. 6), indicating that BETd-260-mediated apoptosis resulted in inhibition of tumor cell proliferation.

We next investigated the anti-OS efficacy of BETd-260 in a subcutaneous OS xenograft mouse model of the MNNG/HOS cell line. Administration of BETd-260 intravenously at 5 mg/kg three times a week for 3 weeks resulted in significant tumor growth inhibition (~94% TGI) (Fig. 5c). After 3 doses, tumors became smaller than those on the day treatment initiated (from 164 to 155 mm^3^), and this partial reduction of tumor volumes lasted to 69 days after the cessation of the treatment (Fig. 5c). On day 121, when the study was terminated, the tumor volume reached 1540.1 ± 612.1 mm^3^ in the vehicle group (Fig. 5d). In striking contrast, tumor volume in BETd-260-treated group was only 246.9 ± 316.7 mm^3^ on the same day, and 5 out of 7 tumors were still smaller than those on the day when treatment started (Fig. 5d). This suggests that efficacy of the BETd-260 treatment is durable. During the treatment, mice experienced no statistically significant weight loss when compared with the animals in the control group, and did not show other signs of toxicity (Fig. 5e).

### BETd-260 induces BET degradation, triggers massive apoptosis in vivo, and inhibits tumor growth in a PDX xenograft mouse model

**Fig. 6:**
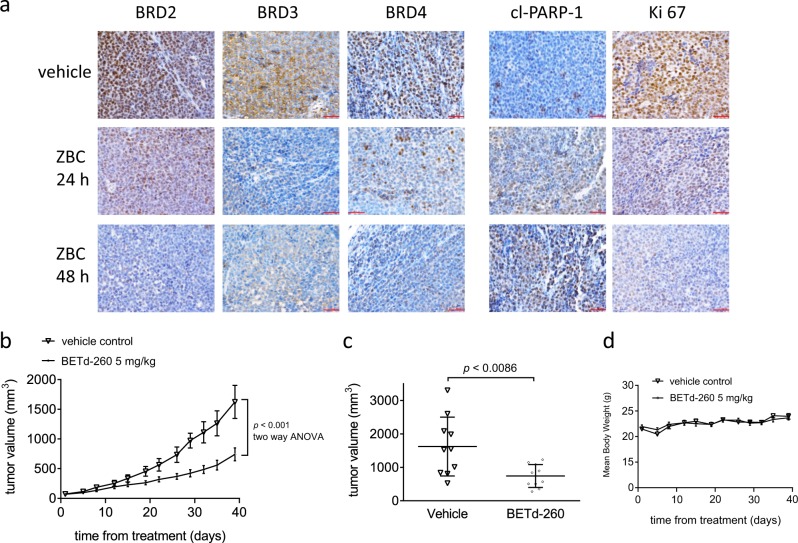
BETd-260 induces BET degradation, triggers apoptosis in vivo, and inhibits tumor growth in PDX xenografts in mice. **a** NOD SCID mice bearing PDX xenograft tumors were treated with a single intravenous dose of 5 mg/kg BETd-260. Two to three mice were sacrificed and tumor tissue was harvested at each different time points. The expression of BRD2, BRD3, BRD4, cleaved PARP-1, and Ki67 was examined by immunohistochemistry staining. **b**–**d** NOD SCID mice bearing xenograft tumors were treated with BETd-260 or vehicle control intravenously three times per week for 3 weeks. Tumor sizes and body weights were measured every 2–3 days. Data are mean ± SEM (*n* = 7–8). **b** Tumor values were plotted with Prism 6 software. **c** Scatter plots represent the tumor volumes at the end of each study. **d** Body weights were plotted with Prism 6 software. *p* values between each treated and the vehicle control group were determined using two-way ANOVA. *p < 0.05; **p < 0.01; ***p < 0.001; ****p < 0.0001

PDX tumors may better reflect the patients’ response to treatment in preclinical study because these tumors largely retain the original tissue histology and cellular morphology^19^. We next evaluated the anti-OS activity of BETd-260 in a subcutaneous OS PDX mouse model. PD study by IHC staining showed that a single intravenous dose of BETd-260 (5 mg/kg) markedly reduced the levels of BET proteins, and led to a large amount of cells positive for cleaved PARP-1, accompanied by inhibition of Ki67-positive staining in the PDX tumors (Fig. 6a). Moreover, intravenous BETd-260 at 5 mg/kg (three times per week for 4 weeks) effectively inhibited this PDX tumor growth with 57% TGI (Fig. 6b). When the tumor volume reached 1623.4 ± 881.9 mm^3^ in the vehicle group on day 39, we terminated the study. On the same day, the average tumor volume in the BETd-260 group was only 740.9 ± 343.4 mm^3^ (Fig. 6c). No significant weight loss or apparent toxicity was observed (Fig. 6d).

## Discussion

Targeting oncogenic protein with small molecule PROTAC degraders has drawn increasing attention over the past few years^12–15,20–22^. Recent studies showed that BET PROTACs elicited strong anticancer activity in several types of hematological cancers and in certain types of solid cancers^12–15^. In the present study, we investigated whether BET PROTAC degraders could be used to treat OS. Our data showed that BET PROTAC BETd-260 potently suppressed the cell viability in a panel of four OS cell lines. Compared with BET inhibitors HJB-97 and JQ1, the biological activity of BETd-260 increased over 1000 times. Importantly, the BET PROTAC was capable of completely abrogating the cell viability at low nanomolar concentrations in the sensitive OS cell lines, indicating that the BET PROTAC implemented its anti-OS activity predominately through a cytotoxic, cell-killing effect.

In this study, we demonstrated that BETd-260 was capable of efficiently promoting apoptosis in OS cells. We reached this conclusion through three lines of evidence. First, BETd-260 triggered strong activation of apoptosis signaling and forced most OS cells to undergo apoptosis within hours. Second, BETd-260 treatment also induced apoptosis in OS xenograft tumor tissue. Third, inhibition of caspase activity with pharmaceutical inhibitors almost completely abolished the ability of BETd-260 to induce cell death in OS cells. Previous studies have demonstrated that OS was inherently resistant to apoptosis, and apoptosis resistance of OS cells was correlated with a reduced response to chemotherapy and was associated with poor prognosis^23–25^. Our findings therefore indicated that BET PROTACs could potentially be used to overcome apoptosis resistance in OS cells.

Apoptosis was orchestrated by the interaction of the anti- and pro-apoptotic Bcl-2 family members^26–28^. Previous studies have found that high expression of anti-apoptotic Bcl-2 family members Bcl-xl and Mcl-1 was the primary reason for apoptosis resistance in OS cells. For instance, Pignochino et al. reported that Mcl-1 was highly expressed in 84% of OS specimens and caused OS cells’ resistance to Sorafenib-mediated apoptosis^28^. Wang et al. found that Bcl-xL expression was significantly higher in osteosarcoma tissues than in corresponding non-tumor tissues, and the expression levels of Bcl-xL were correlated with resistance of OS cells to chemo- or radiotherapy-induced apoptosis^29^. Given that the expression of Bcl-xl and Mcl-1 was stringently maintained by BRD4 in other cancer cells^7,30^, we assumed that BETd-260 might also impact the expression of these two anti-apoptotic Bcl-2 family members in OS cells. We confirmed our assumption by analyzing the effect of BETd-260 on the expression of these Bcl-2 members by western blotting and RT-PCR assays. Notably, the reductions of Bcl-xl and Mcl-1 mRNA and protein levels appeared as early as the 1 h time point, indicating that the expression of these two anti-apoptotic Bcl-2 family members was highly dependent on the regulation of BET proteins. Moreover, the reductions were much earlier than PARP-1 cleavage (14 h), suggesting that inhibition of Bcl-xl and Mcl-1 contributed essentially to BETd-260-mediated apoptosis in OS cells.

Recent studies showed that targeting BET proteins also increased the level of pro-apoptotic Bcl-2 family members. For instance, Peirs et al. found that BET inhibitor JQ1 triggered apoptosis signaling in T-cell acute lymphoblastic leukemia by upregulation of Bim^31^. In this study, we also paid special attention to the effect of BETd-260 on several pro-apoptotic Bcl-2 family members in OS cells. Our data indicated that the BET degrader induced a dramatic increase of Noxa, a Bcl-2 member with relative modest pro-apoptotic function, but had no or little effect on the level of Bim and Bad, two Bcl-2 members with strong pro-apoptotic function in OS cells^32,33^.

Taken together, our results suggest that BET degrader BETd-260 has differential role in regulation of the expression of Bcl-2 family proteins in OS cell lines. BETd-260 has no effect on the expression of the direct activator Bim and the complementary sensitizer Bim^32,33^. In contrast, this degrader robustly and rapidly increases the expression of the sensitizer Noxa, and also reduces the level of Mcl-1 and Bcl-xl, two critical anti-apoptotic Bcl-2 family members to negligible levels within a few hours. The consequence of the effects on Mcl-1, Bcl-xl and Noxa overwhelmingly alter the ratio of pro-apoptotic over anti-apoptotic Bcl-2 family members toward apoptosis.

Noxa is a known target of the p53 tumor suppressor protein. However, our data showed that BETd-260 induced an increase of Noxa not only in p53 wild-type SJSA-1 cells, but also in p53 mutated MNNG/HOS cells, and even in p53-deficient Saos-2 cells. Moreover, p53 level remained largely unchanged during 24-h treatment in SJSA-1 and MNNG/HOS cell lines. These observations suggested that p53 might not be involved in BETd-260-induced increase of Noxa in OS cells. A recent study showed that miR-1271-5p, a c-Myc-driven microRNA, inhibited Noxa protein production by binding to the 3′-UTR of Noxa mRNA^34^. As c-Myc was a critical target of BETd-260 in the OS cells, it is likely that the suppression of c-Myc-miR-1271-5p axis by BETd-260 indirectly leads to the increase of NOXA in OS cells.

Two xenograft OS models allowed us to investigate efficacy of BETd-260 in treatment of OS. Our results demonstrate that BETd-260 strongly inhibits tumor growth in the MNNG/HOS cell-line-derived xenografts in mice. Remarkably, most tumors noticeably shrink after a very short period of therapy (2–3 treatments), even at a lower dosage (5 mg/kg) of BETd-260. This exceptional activity is in striking contrast to the poor-to-modest efficacy produced by treatment with JQ1 alone in human OS cell line-derived xenografts, as reported previously^9,11^. This finding suggests that complete depletion of BET proteins has much stronger efficacy than does sole inhibition of histone binding by BET inhibitors in OS treatment. Treatment with BETd-260 also considerably inhibited the tumor growth in the PDX xenograft tumors, presenting clinically relevant information for using BET PROTAC in OS treatment. Importantly, the dose schedule, 5 mg/kg, three times per week for 3 weeks, was well tolerated in both models. The pharmacodynamics assay by western blotting and immunohistochemistry assays revealed that one dose of BETd-260 efficiently and rapidly removes three BET proteins, and results in massive apoptosis and strong inhibition of proliferation in OS tumor tissues. These data confirm the BETd-260-induced mechanisms of action.

The present study investigates the anticancer activity of a BET PROTAC in OS. To the best of our knowledge, for the first time, we provide evidence that BET PROTACs display a robust apoptotic effect in vitro and in vivo in OS cells, and dramatically inhibit the growth of OS xenografts in mice. Overall, these preliminary findings suggest that targeting BET proteins with PROTAC molecules may have strong potential therapeutic implications in the treatment of human OS.

## Materials and methods

### Cell lines and agents

Human OS Saos-2, MNNG‐HOS, MG‐63, and SJSA-1 cell lines were purchased from the China Center for Type Culture Collection (Wuhan, China) and maintained in RPMI 1640 (HyClone/Thermo Fisher Scientific, Beijing, China) supplemented with 10% heat-inactivated fetal bovine serum (Hangzhou Sijiqing Biological Engineering Materials Co., Ltd, Hangzhou, China). Cells were cytogenetically tested and authenticated before being frozen. Each cell line was maintained in culture for a maximum of 8 weeks after thawing from frozen.

BETd-260 (purity: 99.35%) and HJB-97 were gifts from Dr. Shaomeng Wang at the University of Michigan. The detail chemical information of BETd-260 and HJB-97 were shown in ref. ^14^. MG-132, MLN4924, JQ1, and three caspase inhibitors obtained from Selleck Chemicals Shanghai (Shanghai, China) were dissolved in dimethyl sulfoxide (DMSO) at a stock concentration of 10 mmol/L and were stored at −20 °C.

### CCK-8 cell viability assay

Cell viability was measured using a Cell Counting Kit-8 (Sigma-Aldrich Shanghai, Shanghai, China) to obtain optical density (OD) values. OS cells were plated in 96-well culture dishes (Costar, Cambridge, MA, USA) at a density of 3000–4000 cells/well in 100 μL of medium. Serial dilutions were generated from a stock solution of compounds to the desired concentrations. All experimental concentrations were replicated in triplicate. Before the desired time points, 10 μL CCK-8 was added. After 2–4 h incubation, the absorbance was measured at 450 nm using a microplate reader. Absorbance percentages for treated cells relative to those of untreated control samples were plotted as a function of drug concentration. Inhibition of cell viability was measured by the percentage of viable cells relative to the control: % inhibition = 100% × ODT/ODC, where ODT is the average OD value of the treated samples and ODC is the average OD value of the control samples.

### Cell death and apoptosis flow cytometry assays

Cell death was quantified by microscopic examination in Trypan blue exclusion assays. An apoptosis assay was performed by staining cells with Annexin-V-FITC/PI and examining apoptosis by flow cytometry with a BD LSR II system (BD Biosciences, Shanghai, China). The assays were performed in duplicate with at least three replications per treatment.

### Western blotting

Western blotting was performed as described previously^35,36^. Cells or tumor tissues were lysed using Radio Immunoprecipitation Assay (RIPA) lysis buffer (PBS containing 1% NP40, 0.5% Na-deoxycholate, and 0.1% SDS) supplemented with 1 μmol/L phenylmethylsulfonyl fluoride and 1 protease inhibitor cocktail tablet per 10 mL on ice. Lysate protein concentrations were determined using the Bio-Rad protein assay kit according to the manufacturer’s instructions. Proteins were electrophoresed onto 4–20% SDS-PAGE gels and transferred onto polyvinylidene difluoride membranes. Following blocking in 5% milk, the membranes were incubated with a specific primary antibody, washed, and incubated with horseradish peroxidase–linked secondary antibody (GE Healthcare, Beijing, China). Signals were visualized with chemiluminescent horseradish peroxidase antibody detection reagent (Denville Scientific, Guangzhou, China).

The antibodies against BRD2 (A302-583A, 1:5000), BRD3 (A302-368A, 1:5000), and BRD4 (A700-005, 1:1000) were purchased from Bethyl Laboratories (Shanghai, China). PARP-1 (#9532, 1:1000), p53 (#9282, 1:1000), c-Myc (#9402, 1:1000), Bim (#2819, 1:1000), Bad (#9292, 1:500), Bax (#2774, 1:1000) and Bak #3814, 1:1000) were purchased from Cell Signaling Technology (Shanghai, China). Caspase-9 (sc-56076, 1:500), Caspase-3 (sc-7148, 1:250), Bcl-2 (sc-7382, 1:250), Mcl-1 (S-19) (sc-819, 1:1000), Bcl-xl (C-20) (sc-325, 1:1000), Noxa (114C307) (sc-56169, 1:250) and tubulin (sc-5724, 1:500) were purchased from Santa Cruz Biotechnology (Shanghai, China).

### RNA interference

siRNAs oligos against Noxa, Bax and Bak were purchased from GE Dharmacon (Shanghai, China). Non-targeting control siRNAs (siCtl) were purchased from Qiagen (Shanghai, China). The siRNA transfections (30 pmol/L) were performed using Lipofectamine RNAiMax transfection reagent (Invitrogen, Shanghai, China).

### Immunohistochemistry (IHC)

Tumor tissues were obtained from tumor-bearing mice treated with one dose of BETd-260 5 mg/kg or vehicle control. The following antibodies were used for IHC: BRD2 (A302-583A, 1:250), BRD3 (A302-368A, 1:250), BRD4 (A700-005, 1:100) from Bethyl Laboratories (Shanghai, China); cleaved PARP-1 (Asp214) (5625, 1:100) and Ki67 (8D5) (9449, 1:500) from Cell Signaling Technology (CST, Shanghai, China). IHC was performed following a standard protocol. Briefly, the 5-µm sections were de-paraffinized with xylene, rehydrated in graded concentrations of ethanol, and boiled in antigen retrieval buffer (Abcam, Shanghai, China) in a microwave oven for 5 min. Slides were incubated with specific primary antibodies at room temperature for 2 h. After incubation, the slides were washed three times with PBS and incubated with horseradish peroxidase (HRP)-conjugated antibody (Invitrogen, Shanghai, USA) at room temperature for 30 min, followed by incubation with ABC (avidin-biotin complex, Vectorlabs, Shanghai, China) for 30 min and visualization by the addition of 3,3′-diaminobenzidine tetrahydrochloride (DAB) reagent (Dako Diagnostics Co., Ltd., Shanghai). Sectioned tissues were counterstained with hematoxylin, dehydrated through a graded series of alcohol into xylene, and mounted under glass coverslips. Images of stained slides were captured using a standard light microscope.

### Animal studies

MNNG/HOS cells (5 million cells per tumor) suspended in 0.1 mL of Matrigel were injected subcutaneously into the flanks of 6-week-old BALB/c mice (Charles River, Beijing, China). PDX tumor masses (300–1000 mg per mouse) were implanted subcutaneously into the flanks of 6-week-old NOD SCID mice (Charles River, Beijing, China). For the pharmacodynamics investigation, when tumors reached 100–200 mm^3^, mice (each mouse bearing on tumor) were treated with vehicle control or a single dose of BETd-260; were euthanized at 1, 4, 8, and 24 h time points; and tumor tissue was harvested. For in vivo efficacy studies, when tumors reached about 100 mm^3^, mice were randomized into treated or control groups. For MNNG/HOS model, there were seven mice in the control group and also seven mice (each mouse bearing one tumor) in the BETd-260-treated group, respectively. For PDX model, there were 10 mice in the control group and also 10 mice (each mouse bearing on tumor) in the BETd-260-treated group, respectively. BETd-260 (5 mg/kg) or vehicle control (10% PEG400: 3% Cremophor: 87% PBS, 2% TPGS: 98% PEG200) was administrated at the dose and for the duration indicated. Tumor sizes and animal weights were measured 1–3 times per week. Tumor volume (mm^3^) = (length × width^2^)/2. Tumor growth inhibition was calculated as TGI = (Vc−Vt)/(Vc−Vo) × 100, where Vc, Vt are the median of control and treated groups at the end of the study and Vo at the start. All in vivo studies were performed under an animal protocol approved by the University Committee on Use and Care of Animals of the Zhengzhou University. All animals received humane care according to the criteria outlined in the “Guide for the Care and Use of Laboratory Animals Chinese Version” (2006).

### Statistical analysis

Each experiment was repeated independently three times. The results were displayed as the mean ± SE unless otherwise specified, and were compared with an unpaired *t*-test or ANOVA using GraphPad Prism 6 software. *p* < 0.05 was deemed significant.

## Supplementary information

SUPPLEMENTAL Figure 1

SUPPLEMENTAL Figure 2

SUPPLEMENTAL Figure 3

SUPPLEMENTAL Figure 4

SUPPLEMENTAL Figure 5

SUPPLEMENTAL Figure 6

Author contribution form
