# Supplementary figures and images for: PROTAC induced-BET protein degradation exhibits potent anti-osteosarcoma activity by triggering apoptosis
Source: Cell Death Dis. 2019 Oct 25;10(11):815. doi: 10.1038/s41419-019-2022-2 (PMC6814818; doi:10.1038/s41419-019-2022-2)

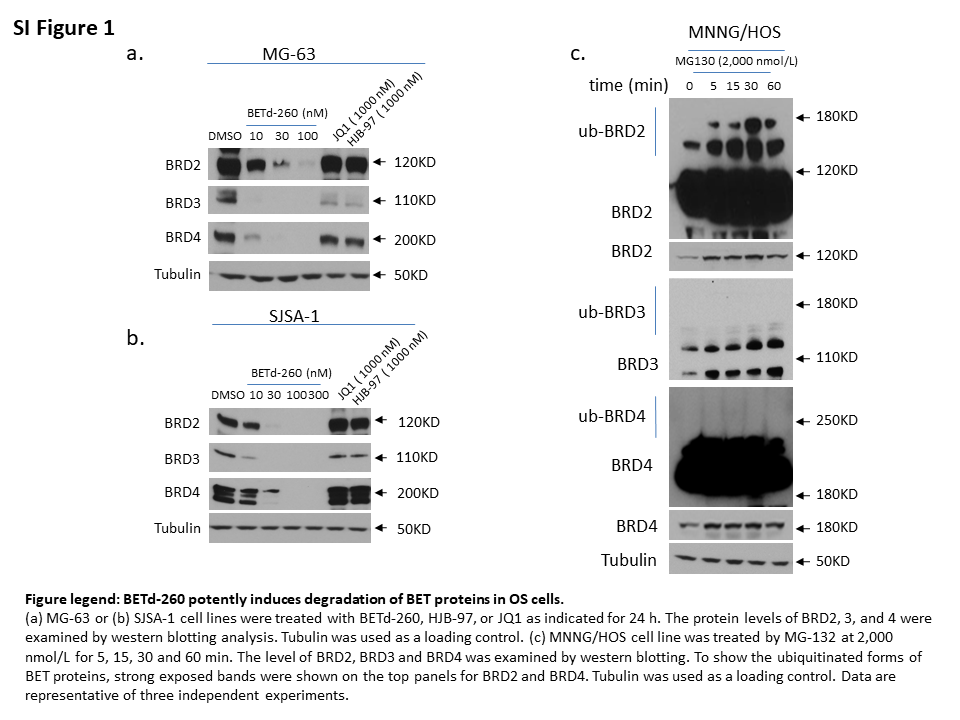

Supplement: Supplementary file 1 — SUPPLEMENTAL Figure 1 [file 41419_2019_2022_MOESM1_ESM.tif]

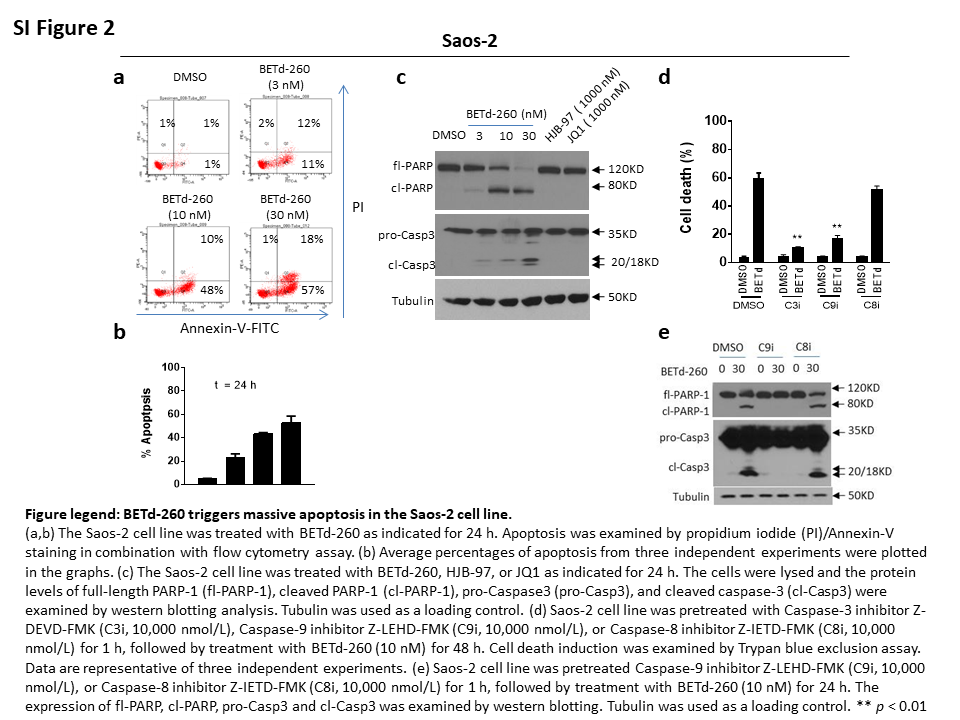

Supplement: Supplementary file 2 — SUPPLEMENTAL Figure 2 [file 41419_2019_2022_MOESM2_ESM.tif]

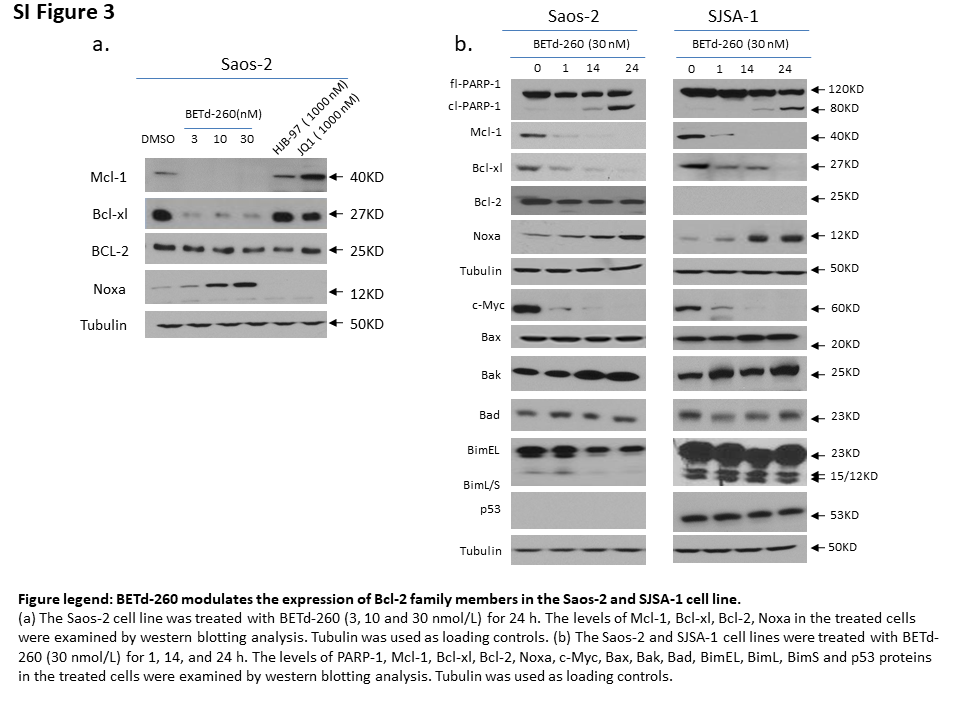

Supplement: Supplementary file 3 — SUPPLEMENTAL Figure 3 [file 41419_2019_2022_MOESM3_ESM.tif]

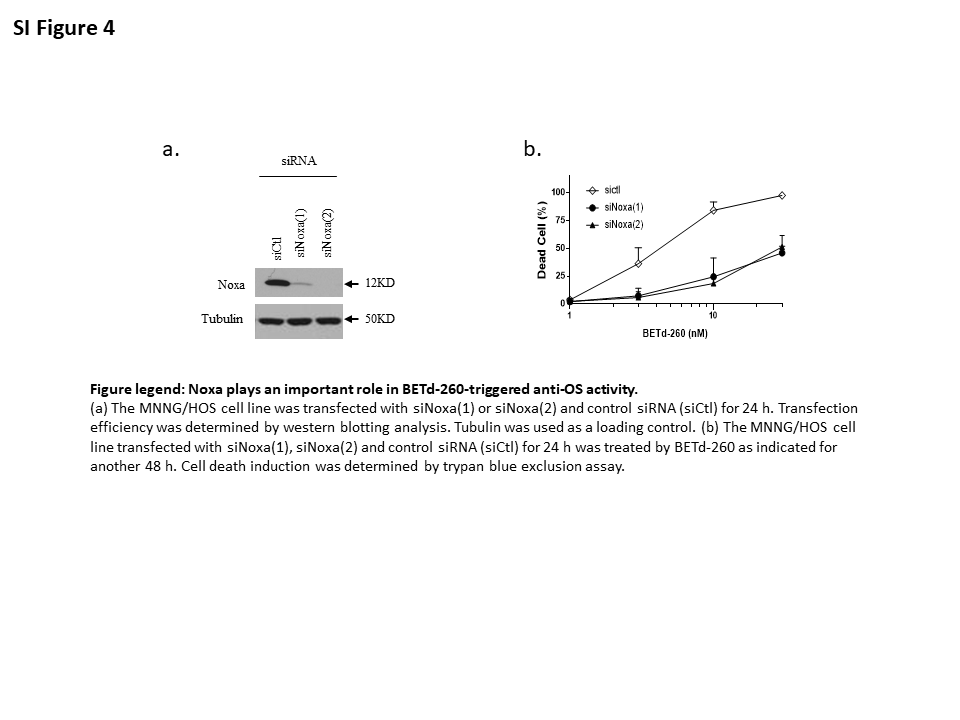

Supplement: Supplementary file 4 — SUPPLEMENTAL Figure 4 [file 41419_2019_2022_MOESM4_ESM.tif]

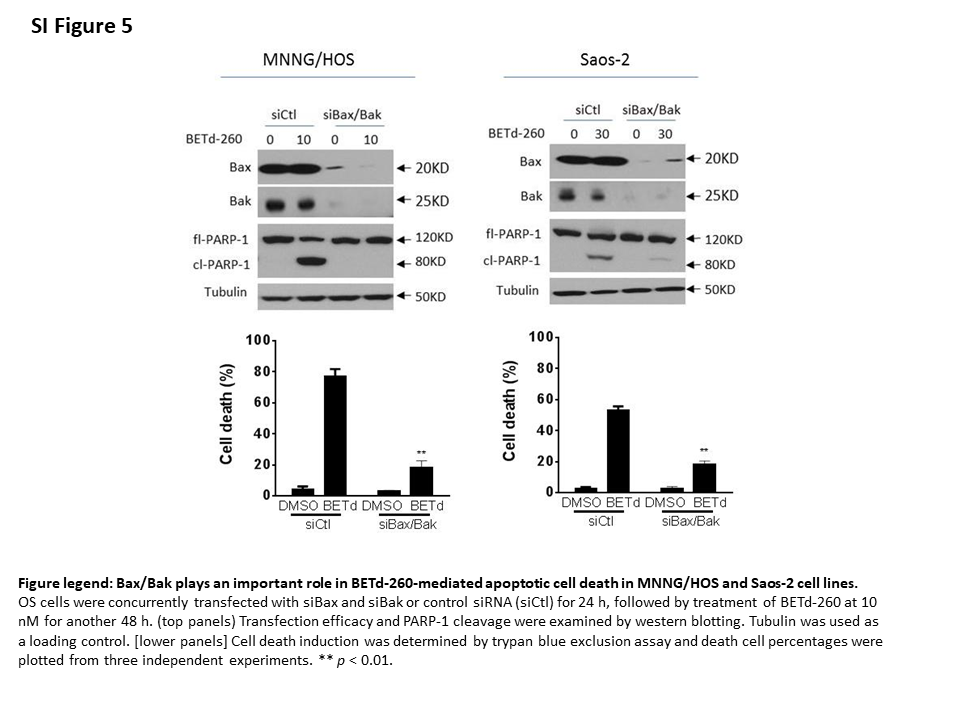

Supplement: Supplementary file 5 — SUPPLEMENTAL Figure 5 [file 41419_2019_2022_MOESM5_ESM.tif]

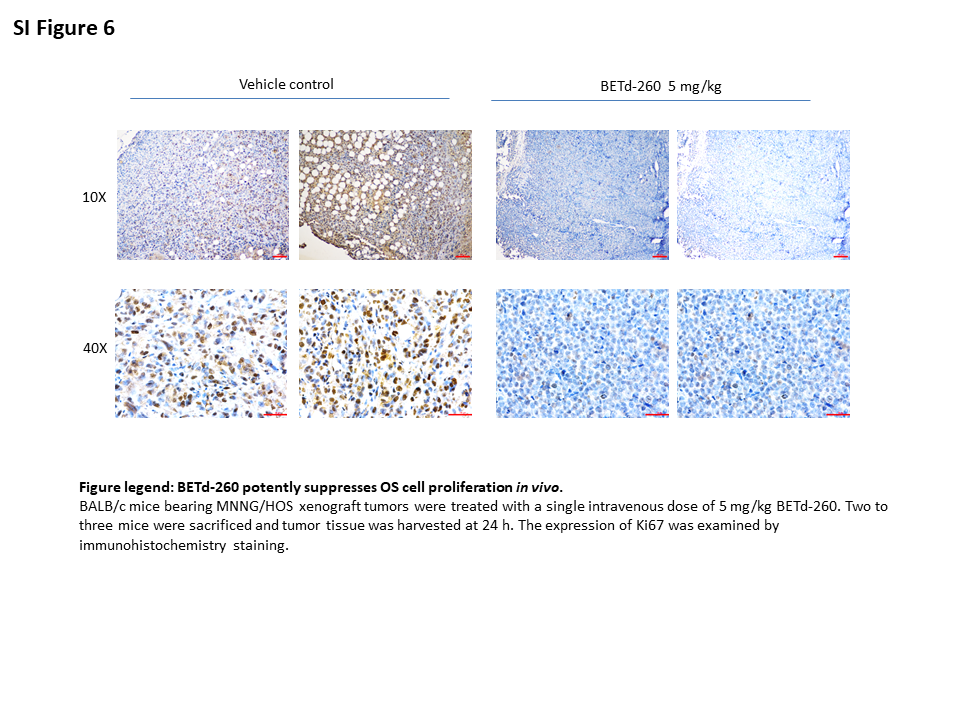

Supplement: Supplementary file 6 — SUPPLEMENTAL Figure 6 [file 41419_2019_2022_MOESM6_ESM.tif]
