# Supplementary material for: PROTAC induced-BET protein degradation exhibits potent anti-osteosarcoma activity by triggering apoptosis
Source: Cell Death Dis. 2019 Oct 25;10(11):815. doi: 10.1038/s41419-019-2022-2 (PMC6814818; doi:10.1038/s41419-019-2022-2)
Supplement: Supplementary file 7 — Author contribution form [file 41419_2019_2022_MOESM7_ESM.pdf]

DECLARATION OF CONTRIBUTIONS TO ARTICLE

ADMC

Manuscript Number:	Journal Name:	
CDDIS-19-1973R	*Cell Death & Disease*	(the 'Journal')

Proposed Title of the Contribution:

PROTAC induced-BET protein degradation exhibits potent anti-osteosarcoma activity by triggering apoptosis (the 'Contribution)

Author(s):

Chengcheng Shi, Huapeng Zhang, Penglei Wang, Kai Wang, Denghui Xu, Haitao Wang, Li Yin, Shuijun Zhang, Yi Zhang (the 'Authors')

For all *CDDis* articles, each person named as an author in the published version must be able to show he or she has contributed substantially to the article.

Authorship credit should be based on 1) substantial contributions to conception and design, acquisition of data, or analysis and interpretation of data; 2) drafting the article or revising it critically for important intellectual content; and 3) final approval of the version to be published. Authors should meet conditions 1, 2 and 3.

Any person who cannot be shown to have made a substantial contribution to the article cannot be listed as an author in the final version. The name of any person who is deemed to have made a minor contribution can, however, appear in the Acknowledgments section of the article.

Please complete the table below to indicate the contributions of all named authors to the manuscript.

Author Full Name:	Specification of Contribution to the Manuscript:
Chengcheng Shi	CS design of the experiments, carried out most of the experiments and drafted the manuscript
Huapeng Zhang	HZ design of the experiments, carried out most of the experiments and drafted the manuscript
Penglei Wang	PW performed the PDX experiment
Kai Wang	KW contributed to the animal experiments and some molecular experiments
Denghui Xu	DX carried out some molecular experiments
Haitao Wang	HW carried out flow cytometry and collected the data
Li Yin	LY carried out flow cytometry and collected the data
Shuijun Zhang	SZ participated in the design of the study and revised the manuscript
Yi Zhang	YZ designed the study and revised the manuscript

Please complete the table below to indicate the contributions of all named authors to the figures.

Figure 1:

CS and HZ generated the data and assembled the figure.

Figure 2:

CS generated the WB data and assembled the figure.

Figure 3:

HZ generated the cell death and flow flow cytometry data and prepared panel a, b, d. CS generated the WB data and assembled the figure.

Figure 4:

SC and DX generated the WB data and assembled the panel a, b, c. HZ generated the RNA interference data.

Figure 5:

HZ, KW and DX generated the data, HW and LY generated the immune-histochemistry data and labelled the image, KW assembled the figure.

Figure 6:

PW generated the data, HW and LY generated the immune-histochemistry data and labelled the image, KW assembled the figure.

Signed for and on behalf of the Author(s):	Print Name:	Date:
Yi Zhang	Yi Zhang	September 13, 2019
